# Supplementary material for: Construction of long non-coding RNA- and microRNA-mediated competing endogenous RNA networks in alcohol-related esophageal cancer
Source: PLoS One. 2022 Jun 15;17(6):e0269742. doi: 10.1371/journal.pone.0269742 (PMC9200351; doi:10.1371/journal.pone.0269742)
Supplement: S1 Table — (DOCX) [file pone.0269742.s001.docx]

**S1 Table. The clinical features related miRNAs.**

| Comparisons | Related miRNAs | |
| --- | --- | --- |
|  | Up-regulated | Down-regulated |
| Age at diagnosis (≥ 60 vs.< 60) | *hsa-mir-3648*, *hsa-mir-1269*, *hsa-mir-205*, *hsa-mir-1293*, *hsa-mir-944* | *hsa-mir-7*, *hsa-mir-577* |
| Clinical M (MX vs. M0) | *hsa-mir-3648*, *hsa-mir-767*, *hsa-mir-944* | - |
| Clinical N (N3+N2 vs. N0+N1) | *hsa-mir-105-2*, *hsa-mir-767*, *hsa-mir-675* | - |
| Clinical T (T3 + T4 vs. T1 + T2) | *hsa-mir-105-2*, *hsa-mir-767* | *hsa-mir-1269, hsa-mir-675, hsa-mir-944, hsa-mir-205* |
| Clinical stage (III+IV vs. I+II) | *hsa-mir-205*, *hsa-mir-508*, *hsa-mir-3662*, *hsa-mir-412* | *hsa-mir-7-3*, *hsa-mir-1269*, *hsa-mir-421, hsa-mir-675* |
| Neoplasm histologic grade (G3+G4 vs. G1+G2) | *hsa-mir-508*, *hsa-mir-1293*, *hsa-mir-944*, *hsa-mir-205* | *hsa-mir-7-2*, *hsa-mir-7-3*, *hsa-mir-1304*, *hsa-mir-483*, *hsa-mir-2110*, *hsa-mir-105-2*, *hsa-mir-877*, *hsa-mir-767* |
| Tumor status (Yes vs. No) | *hsa-mir-508*, *hsa-mir-1293*, *hsa-mir-205*, *hsa-mir-944* | *hsa-mir-7-3*, *hsa-mir-7-2*, *hsa-mir-2110*, *hsa-mir-877*, *hsa-mir-577* |
| Smoking status (Yes vs. No) | *hsa-mir-133a-1*, *hsa-mir-767*, *hsa-mir-1-2*, *hsa-mir-105-2*, *hsa-mir-1269* | *hsa-mir-627*, *hsa-mir-1249*, *hsa-mir-1293*, *hsa-mir-944*, *hsa-mir-33a*, *hsa-mir-135b*, *hsa-mir-205* |

hsa, *Homo sapien*s; miR, microRNA.
