# Supplementary material for: Construction of long non-coding RNA- and microRNA-mediated competing endogenous RNA networks in alcohol-related esophageal cancer
Source: PLoS One. 2022 Jun 15;17(6):e0269742. doi: 10.1371/journal.pone.0269742 (PMC9200351; doi:10.1371/journal.pone.0269742)
Supplement: S2 Table — (DOCX) [file pone.0269742.s002.docx]

**S2 Table. The clinical features related mRNAs.**

| Comparisons | Related mRNAs | |
| --- | --- | --- |
|  | Up-regulated | Down-regulated |
| Age at diagnosis (≥ 60 vs.<60) | *KRT5*, *LY6D*, *PKP1*, *CALML3*, *KRT16*, *FGFBP1*, *TRIM29*, *DSG3*, *KRT6A*, *AHNAK2*, *KRT6B*, *FOSB* | *SVIP*, *GATM* |
| Clinical M (MX vs. M0) | *CST1*, *FOSB* | *CA2*, *GATM* |
| Clinical N (N3+N2 vs. N0+N1) | *-* | *MMP3*, *RHCG*, *MMP1*, *DEFB1* |
| Clinical T (T3 + T4 vs. T1 + T2) | *KRT6B*, *FGFBP1*, *PKP1*, *KRT6A*, *TP63*, *SPRR1B*, *RHCG*, *KRT6C*, *DSG3*, *GJB6*, *ALDH3A1*, *CALML3*, *SPRR1A*, *SPRR3*, *SPRR2D*, *SPRR2A****,*** *KRT13* | *S100A8*, *S100A2*, *KRT16*, *KRT14*, *CSTA*, *KRT5*, *CLCA2, LYPD3*, *CNFN* |
| Clinical stage (III+IV vs. I+II) | *KRT5*, *KRT13*, *IGLL5*, *LCP1* | *MMP1*, *S100P* |
| Neoplasm histologic grade (G3+G4 vs. G1+G2) | *KRT6A, KRT5, DSG3, SPRR1A, LY6D, KRT6B, CALML3, FGFBP1, KRT13*, *GJB6*, *WNT7B*, *AKR1B10*, *ALDH3A1* | *GDF15, CD248, PIGR, MMP1* |
| Tumor status (Yes vs. No) | *S100A14*, *KRT17*, *KRT6A*, *KRT16*, *SLPI*, *AGR2*, *RHCG*, *SPRR1B*, *COL17A1*, *ITGB6*, *CXCL17*, *GJB6*, *SPRR2D* | *GPC4*, *CST1* |
| Smoking status (Yes vs. No) | *RHCG*, *SPRR2A*, *SPRR1A*, *KRT16*, *GJB6*, *CNFN*, *KRT6B*, *KRT6C* | *KRT6A*, *SPRR1B*, *CEACAM6*, *KRT13* |
