# Supplementary material for: Construction of long non-coding RNA- and microRNA-mediated competing endogenous RNA networks in alcohol-related esophageal cancer
Source: PLoS One. 2022 Jun 15;17(6):e0269742. doi: 10.1371/journal.pone.0269742 (PMC9200351; doi:10.1371/journal.pone.0269742)
Supplement: S3 Table — (DOCX) [file pone.0269742.s003.docx]

**S3 Table. The clinical features related lncRNAs.**

| Comparisons | Related lncRNAs | |
| --- | --- | --- |
|  | Up-regulated | Down-regulated |
| Age at diagnosis (≥ 60 vs.<60) | *HOXA-AS3*, *MIR205HG* | *AFAP1-AS1* |
| Clinical M (MX vs. M0) | *MIR205HG* | *HOXB-AS3* |
| Clinical N (N3+N2 vs. N0+N1) | *-* | *CCAT1* |
| Clinical T (T3 + T4 vs. T1 + T2) | *HOXB-AS3* | *RNU11*, *HOXA-AS3*, *MIR205HG*, *MYLK-AS1*, *H19*, *SCARNA2* |
| Clinical stage (III+IV vs. I+II) | *SNHG10*, *SCARNA2*, *H19*, *MIR205HG* | *RNU11* |
| Neoplasm histologic grade (G3+G4 vs. G1+G2) | *HOXB-AS3*, *AFAP1-AS1*, *RNU11* | *MIR205HG* |
| Tumor status (Yes vs. No) | *MIR205HG* | *HOXB-AS3*, *MCF2L-AS1*, *AFAP1-AS1* |
| Smoking status (Yes vs. No) | *MYLK-AS1*, *CCAT1*, *MIR205HG*, *RBM12B-AS1* | *MFI2-AS1* |

lncRNAs, long non coding RNAs.
