# Supplementary material for: Construction of long non-coding RNA- and microRNA-mediated competing endogenous RNA networks in alcohol-related esophageal cancer
Source: PLoS One. 2022 Jun 15;17(6):e0269742. doi: 10.1371/journal.pone.0269742 (PMC9200351; doi:10.1371/journal.pone.0269742)
Supplement: Attachment — Submitted filename: Response to reviewers.docx [file pone.0269742.s005.docx]

Dear editor,

We would like to extend our most sincere gratitude to the reviewers' comments on our manuscript entitled "**Construction of long non-coding RNA-and microRNA-mediated competing endogenous RNA networks in alcohol-related esophageal cancer** ". Those comments are all valuable and very helpful for revising and improving our paper, as well as the essential guiding significance to our researches. We have revised the comments extensively and have made the corrections in order to meet with approval. The leading corrections in the paper and the response to the reviewers' comments are as follows.

We appreciate for Editors/Reviewers' warm work earnestly and hope that the correction will meet with approval.

Once again, thanks for the comments and suggestions.

Sincerely,

Quan Du

**A point-by-point response to editor’s and reviewer’s comments**

**Academic Editor**

Journal Requirements:

Please review your reference list to ensure that it is complete and correct. If you have cited papers that have been retracted, please include the rationale for doing so in the manuscript text, or remove these references and replace them with relevant current references. Any changes to the reference list should be mentioned in the rebuttal letter that accompanies your revised manuscript. If you need to cite a retracted article, indicate the article’s retracted status in the References list and also include a citation and full reference for the retraction notice.

**Response:** Thank you for your valuable comment. We have checked all the references, and no retracted papers are cited in our manuscript.

In this revision, no references are deleted, and doi number are added for references if their doi number are available.

**Reviewer #1**

The authors have addressed all my comments. I think the revised manuscript is acceptable for publication.

**Response:** Thank you for your recommendation for our manuscript.

**Reviewer #2**

There are many mistakes in the information of references. The authors should carefully check and revise them. For example, the reference [76] has no journal name and the author’s information is incomplete; the reference information of [77] should be 2020, 191: 105261, and the reference information of [78] should be 2021, 22(6): 1-27 with DOI: 10.1093/bib/bbab286

**Response:** Thank you for your valuable comment. We have checked all the references to correct the mistakes.

The revised references 76-77 are as follows:

76.	Zhang L, Yang P, Feng H, Zhao Q and Liu H: Using Network Distance Analysis to Predict lncRNA-miRNA Interactions. Interdiscip Sci. 2021. 13 (3):535-545. doi:10.1007/s12539-021-00458-z

77.	Liu H, Ren G, Chen H, Liu Q and Zhao Q: Predicting lncRNA-miRNA interactions based on logistic matrix factorization with neighborhood regularized. Knowledge-Based Systems. 2020. 191:105261. doi:10.1016/j.knosys.2019.105261

78.	Wang CC, Han CD, Zhao Q and Chen X: Circular RNAs and complex diseases: from experimental results to computational models. Briefings in bioinformatics. 2021. 22 (6):1-27. doi:10.1093/bib/bbab286
